# Penile Curvature Incidence in Hypospadias: Can It Be Determined?

**DOI:** 10.1155/2011/813205

**Published:** 2011-10-09

**Authors:** Borko Stojanovic, Marta Bizic, Marko Majstorovic, Vladimir Kojovic, Miroslav Djordjevic

**Affiliations:** Department of Urology, University Children's Hospital, University of Belgrade, Tirsova Street, 11000 Belgrade, Serbia

## Abstract

The aim was to retrospectively determine the real incidence of congenital penile curvature in various forms of hypospadias, in order to indicate intraoperative assessment and correction of curvature. We analyzed 842 patients with hypospadias who underwent surgery from 2003 to 2010, classified into two groups. First group was intraoperatively checked for curvature as a routine procedure, while a curvature in the second group was assessed mostly in severe hypospadias. Results are analyzed using Fisher's and chi-square tests. In total, 238 cases (28.3%) of associated curvature were confirmed. Curvature was significantly more frequent in the first group, regarding hypospadias in general (*P* < 0.01), as well as distal (*P* < 0.05) and midshaft forms (*P* < 0.01). Penile curvature is common figure in hypospadias, including distal types. Intraoperative testing for associated curvature should be considered as a routine procedure in hypospadias repair.

## 1. Introduction

Hypospadias is a congenital defect of the penis resulting in incomplete development of the penile urethra, occurring in approximately 1 in 300 live births. Hypospadias may be accompanied by different genital malformations. The frequency of associated anomalies increases with the severity of hypospadias. Cryptorchidism and inguinal hernia are the most commonly associated anomalies, while curvature of the penis is most common in severe cases of hypospadias. Typically, penile curvature associated with hypospadias is in the ventral direction [1]. It is caused by tethering of the skin, fibrosis, and contracture of the fascial tissue surrounding the urethra, a disproportionately large corpora or a short urethral plate (condition designated as congenital chordee). Associated curvature is diagnosed intraoperatively. The preferred time for surgery is between the ages of 6 and 18 months, before the child develops body image and castration anxiety [2]. Although the patients are not yet adults, when chordee and erectile dysfunction may become apparent, early correction of associated penile curvature will have the positive impact on sexual relationship, confidence, self-esteem, and sexual function in the future [3]. 

Despite weak evidence on the incidence of penile curvature in hypospadias, the literature finds curvature mostly associated with severe hypospadias, without pointing out its incidence in general or appearance in distal hypospadias [4–7]. 

The aim of our retrospective study was to try to determine the real incidence of congenital curvature in various forms of hypospadias, in order to indicate curvature testing as a routine procedure in hypospadias repair.

## 2. Materials and Methods

The study was carried out in the period from 2003 to 2010 at the University Children's Hospital. Surgical protocols and records were used to register every case of hypospadias and potentially associated curvature treated in this period, including both new and redos. In most cases, reoperations were required due to the occurence of urethral fistula. Hypospadias repair surgeries were performed by two groups of surgeons, and their different assessment of curvature was the criteria for classifying patients into two groups. The patients in group one (I) were intraoperatively tested for curvature as a standard procedure performed by first group of surgeons, using artificial erection or pharmacological erection with prostaglandin E1. As for patients in group two (II), associated curvature was only observed during the surgery or checked for only in proximal, severe hypospadias by the second group of surgeons. 

In total, 842 patients with hypospadias, aged 7 months to 24 years, were surgically treated from 2003 to 2010. Mean followup was 48 months (range 14 to 86 months). Different urethroplasty techniques were performed in hypospadias repair. Artificial erection or pharmacological erection induced by prostaglandin E1 (PGE1) was used for intraoperative diagnosis of penile curvature. The artificial erection has disadvantages in being a forced dilatation of the penile erectile tissue, applied for a limited time and difficult to use in cases of severe hypospadias. Artificial erection has to be applied several times during surgery to assess the anomaly and its correction. Using pharmacological erection with PGE1, one injection usually achieves an erection sufficiently prolonged to assess and correct the anomaly. It involves a PGE1 injection intracavernosally on the lateral aspect of the midportion of the slightly stretched penis [8]. 

Any penile angulation greater than 20° was considered as curvature that required surgical correction, for both groups of surgeons. Plication corporoplasty was performed as a standard procedure, giving good and stable results [8]. 

We have analysed the number of patients treated for penile curvature in various types of hypospadias, for both groups. We have also compared scores in two groups, using Fisher's test and chi-square test. The sum of *P* values less than 0.05 is considered statistically significant, and less than 0.01 extremely statistically significant.

## 3. Results and Discussion

### 3.1. Results

During this period, penile curvature was treated as an associated anomaly in 238 cases (28.3%) of 842 patients with hypospadias. Incidence of curvature varied in different types of hypospadias: 23.5% in distal types, 29.4% in midshaft, and 68.3% in proximal forms. Curvature was treated in 9.3% of redo surgeries (Table 1). 

In 512 patients in the first group (I), 184 cases (36%) were treated for associated curvature. Incidence of penile curvature was 31.3% in distal (glanular, coronal, and subcoronal) forms, 40% in midshaft, and 69.4% in proximal (penoscrotal and scrotal) forms of hypospadias. Of 64 reoperations, 6 (9.4%) included correction of curvature (Table 1). There were 330 patients in the second group (II), and 54 of them (16.4%) had a curvature diagnosed; 14% of distal-type cases were accompanied by a curvature, 8.8% of midshaft, and 66.7% of proximal hypospadias. Four of 43 reoperated patients (9.3%) were treated for curvature (Table 1).

Comparing the incidence of curvature in two groups yields a highly significant (*χ*² = 24.229, *P* < 0.01) result (Table 1). Variation in penile curvature incidence between groups is also significant, as was observed in comparing distal hypospadias (*χ*² = 6.222, *P* < 0.05). There are not significant results comparing glanular and coronal, as well as subcoronal hypospadias (Table 1). 

Incidence of curvature in midshaft hypospadias is higher in group one (I) patients (*χ*² = 21.348, *P* < 0.01). Statistically significant result was not obtained when comparing the incidence of proximal hypospadias (*P* = 0.328, *P* > 0.05), particularly penoscrotal and scrotal hypospadias, as well as redo cases (Table 1). 

### 3.2. Discussion

Hypospadias is one of the most common congenital anomalies, with an increasing trend [9]. In Europe, the prevalence of hypospadias in the 1970s and 1980s increased with no obvious explanation. An unexplained doubling in the incidence of hypospadias has been determined in the United States [10]. This genitourinary malformation, particularly proximal forms, involves the spectrum of abnormalities, including ventral curvature of the penis [11].

Penile curvature represents a challenge to classification and particularly to correction. The repair of curvature is imperative, and the search for new and better solutions remains challenging [2]. Penile angulation not only causes potential sexual dysfunction, difficulty and pain during intercourse, or total coital incapacity, but also causes severe psychological problems. Surgical correction is necessary in order to obtain a functionally and cosmetically normal penis, minimizing psychological effects on the child in the future [3]. If associated curvature is unrevealed during hypospadias treatment, this condition will require additional surgery in the future. 

Curvature with hypospadias was defined and discussed, although the real incidence has not yet been determined. According to Baskin et al. [1], in 25% of patients with hypospadias who underwent reconstruction with island flaps (onlay and tubes), they found significant penile bend after releasing the skin and dartos. Samuel et al. [12] reviewed 381 boys with distal hypospadias who underwent surgery in five-year period, reporting that none required correction of chordee. Snodgrass et al. [13] performed the erection test only in the most severe hypospadias with a proximal or perineal division of the spongiosum. They claim the consensus that in distal hypospadias curvature is not a problem and that in proximal hypospadias it should be part of the evaluation during surgery to judge severity and help to choose the ideal technique for repair, considering this approach as standard in hypospadias repair [13]. Long-term experience in treating hypospadias at the University Children's Hospital indicated that this issue should be reevaluated. 

**Figure 1:**
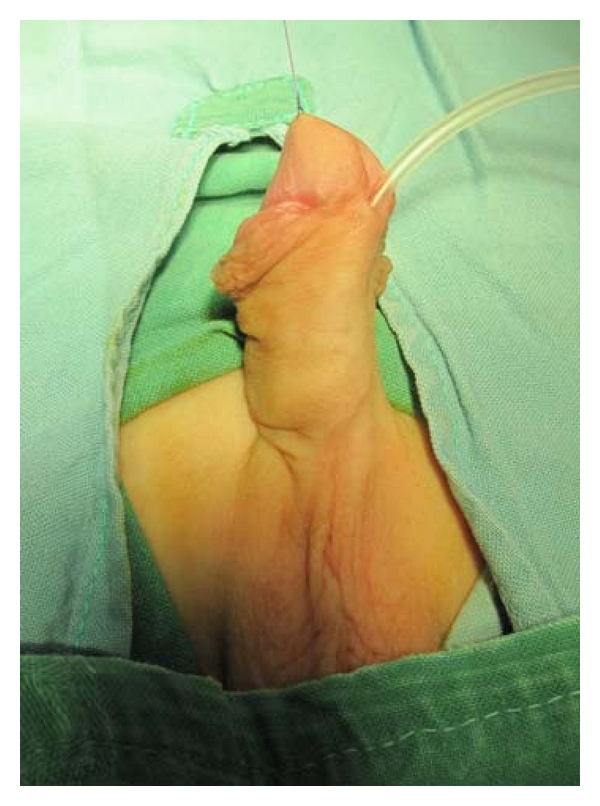
Glanular hypospadias.

**Figure 2:**
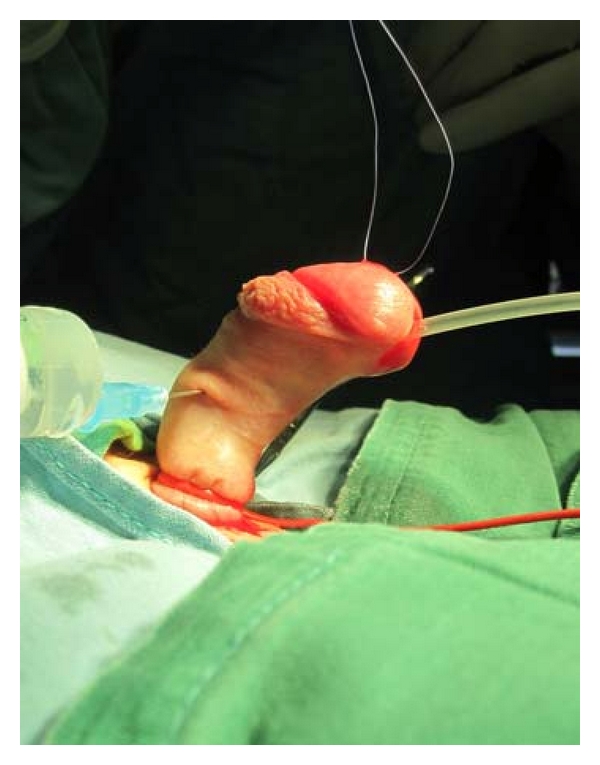
Associated curvature (40°) is confirmed by artificial erection.

The results of our study show high incidence of penile curvature in hypospadias. In approximately 30% of all patients with hypospadias treated from 2003 to 2010, an associated curvature was confirmed. Incidence of curvature varied depending on the form of hypospadias. Incidence diversity between two patient groups is also obvious, both in hypospadias in general, and in midshaft forms and, particularly, distal forms. This significant difference (36% : 16.4%) obviously relates to intraoperative testing for curvature as a standard procedure of the first group of surgeons. Although distal hypospadias is regarded as mild cases, rarely accompanied by penile curvature, our study indicates that even these mild types ought to be checked for this anomaly (Figures 1 and 2). 

Analyzing retrospectively, there is a lack in classification of penile curvature degree in our study. Although we performed correction of every angulation greater than 20°, not every curvature was precisely measured causing the lack of data about exact angle of associated curvature in different hypospadias types. 

Anyhow, hypospadias must not be considered a separate entity, as is often the case in the literature, but should rather be observed integrally with associated anomalies, including penile curvature [14].

## 4. Conclusions

Penile curvature is determined to be common figure in hypospadias, including distal forms. Incidence is statistically analyzed and verified, based on our experience. Intraoperative testing for associated curvature should be considered as a routine procedure in hypospadias repair.

## Figures and Tables

**Table 1:** Type of hypospadias and incidence of associated curvature.

Hypospadias type	Group I curvature/Pts (%)	Group II curvature/Pts (%)	Summary	Ratio group I/group II
Distal				
Glanular	15/36 (41.7%)	4/28 (14.3%)	19/64 (29.7%)	*P* = 0.094, *P* > 0.05
Coronal	11/32 (34.4%)	7/43 (16.3%)	18/75 (24%)	*P* = 0.091, *P* > 0.05
Subcoronal	29/108 (26.9%)	9/72 (12.5%)	38/180 (21.1%)	*χ*² = 1.992, *P* > 0.05
Distal total	55/176 **(31.3%)**	20/143 **(14%)**	75/319 **(23.5%)**	*χ*² = 6.222, *P* < 0.05
Midshaft	89/223 **(40%)**	10/114 **(8.8%)**	99/337 **(29.4%)**	*χ*² = 21.348, *P* < 0.01
Proximal				
Penoscrotal	11/18 (61.1%)	10/16 (62.5%)	*P* = 0.378, *P* > 0.05	*P* = 0.378, *P* > 0.05
Scrotal	23/31 (74.2%)	10/14 (71.4%)	*P* = 0.491, *P* > 0.05	*P* = 0.491, *P* > 0.05
Proximal total	34/49 **(69.4%)**	20/30 **(66.7%)**	54/79 **(68.3%)**	*P* = 0.328, *P* > 0.05
Redo	6/64 (9.4%)	4/43 (9.3%)	10/107 (9.3%)	*χ*² = 0.184, *P* > 0.05
Summary	184/512 **(36%)**	54/330 **(16.4%)**	238/842 **(28.3%)**	*χ*² = 24.229, *P* < 0.01

Group I: patients with hypospadias standardly tested for curvature, treated by the first group of surgeons.

Group II: patients with hypospadias not standardly tested for curvature, treated by the second group of surgeons.
